# Methodological progress note: Hybrid effectiveness‐implementation clinical trials

**DOI:** 10.1002/jhm.12936

**Published:** 2022-08-08

**Authors:** Amanda J. Ullman, Rinad S. Beidas, Christopher P. Bonafide

**Affiliations:** ^1^ School of Nursing, Midwifery and Social Work The University of Queensland Brisbane Queensland Australia; ^2^ Centre for Children's Health Research Children's Health Queensland Hospital and Health Service Brisbane Queensland Australia; ^3^ NHMRC Centre for Wiser Wound Care Griffith University Brisbane Queensland Australia; ^4^ Centre for Clinical Nursing Royal Brisbane and Women's Hospital Brisbane Queensland Australia; ^5^ Perelman School of Medicine University of Pennsylvania Philadelphia Pennsylvania USA; ^6^ Penn Implementation Science Center at the Leonard Davis Institute (PISCE@LDI) University of Pennsylvania Philadelphia Pennsylvania USA; ^7^ Penn Medicine Nudge Unit University of Pennsylvania Health System Philadelphia Pennsylvania USA; ^8^ Section of Hospital Medicine Children's Hospital of Philadelphia Philadelphia Pennsylvania USA

## INTRODUCTION

Randomized controlled trials (RCTs) remain the gold‐standard approach to informing clinical decision‐making and drawing causal inferences.^1^, ^2^ Yet, traditional RCTs have limitations.^1^, ^2^, ^3^, ^4^ Conducting these trials can be painstakingly slow and translation to practice even slower, resulting in meaningful and unacceptable research‐to‐practice gaps. There is often a disconnect in generalizability and performance between the highly controlled clinical trials to the real‐world application of approaches within complex health environments across situational contexts.

Implementation science has emerged as a distinct area of research specifically to bridge the clinical evidence to practice gap. It is the formal study of methods to promote the systematic uptake of research findings and other evidence‐based practices (EBPs) into routine practice, and, hence, improve the quality and effectiveness of health services.^5^ The inclusion of EBPs in the definition means that implementation science as originally conceived was intended to promote interventions that were already proven to be effective. In implementation trials, implementation strategies (such as educational outreach, coaching, facilitation, audit and feedback, and clinical decision support) are tested to determine the best way to promote EBP use.

To reduce the gap in translating effective knowledge from clinical trials, separate effectiveness and implementation trials need not be performed in series, but rather learnings can occur together.^3^ Healthcare systems are complex and understanding the influence of situational context in parallel to understanding the clinical effectiveness is vital, even while the evidence base is actively being developed in clinical trials (individually or cluster‐randomized) or in quasi‐experimental studies. We argue that the most important test of an intervention is its test within the context in which it is meant to be deployed. The hybrid effectiveness‐implementation design offers an innovative and rapid manner to test effectiveness and implementation at the same time.

This methodological progress note has been developed to prepare the Hospital Medicine community to appropriately use hybrid effectiveness‐implementation trials in our own research and ensure, as hybrid trials become more common, we are informed in design attributes, strengths and limitations as educated consumers of health and healthcare delivery literature.

## HYBRID EFFECTIVENESS‐IMPLEMENTATION CLINICAL TRIALS

A hybrid trial has two complementary goals, incorporating an exploration of both *clinical effectiveness* (i.e., does this intervention improve this clinical outcome) and *implementation outcomes* (i.e., how is this intervention applied) within a single trial. Hybrid trials are serious undertakings and require advanced expertise in multiple quantitative and qualitative methods. There is a range of implementation frameworks that exist to guide the design of a robust hybrid trial, including process frameworks, determinant frameworks, and evaluation frameworks.^6^ Below we have listed a few of the most frequently used outcomes within implementation frameworks, with definitions adapted from a landmark paper by Proctor et al.,^7^ and COVID‐specific updates by Pilar et al.,^8^ and encourage the readership to continue their learning via these papers. Please note that below, each outcome is framed relative to an EBP, but could also be framed relative to an implementation strategy (e.g., acceptability of a strategy).

- *Acceptability* is the degree to which those who might be involved or affected by implementation welcome implementation of a new EBP and/or view it as appealing and meeting their approval. It is often measured by survey (e.g., Acceptability of Intervention Measure).^9^
- *Adoption* is the initial decision or action to begin using a new EBP. This outcome can be measured via survey or by analyzing existing administrative or clinical data to determine uptake of the EBP.
- *Fidelity* is the degree to which an EBP is implemented as designed or intended, and may include dimensions of adherence to the original plan, the “dose” of intervention delivered, and the quality of intervention delivery. Fidelity can be measured via checklists, self‐report, and direct observation of the practice.
- *Health equity* involves fair access to treatment or innovation without avoidable or remediable differences among groups of people.^8^
- *Penetration* (also known as *reach*) is the degree to which an EBP is integrated into the desired setting and subunits of that setting (e.g., the percentage of wards in a hospital using an EBP, or the percentage of eligible patients who receive an EBP within a primary care system).
- *Sustainability* is the extent to which an EBP is continued over an extended period of time beyond an initial period of adoption. Associated measures that might promote the sustainment of an EBP are institutionalization and routinization.

In addition to exploring how EBPs can be successfully implemented, hybrid trials can by their design provide insight into the effectiveness of the studied intervention, especially if the clinical outcome was unexpected. Why wasn't the expected benefit seen? What contributed to the difference between expected and obtained results? Hybrid trials can answer broader questions than those related to effectiveness alone. For example, the information was disseminated but not widely accepted or adopted. Or, the intervention was delivered with low fidelity and/or dose. Hybrid trials multiply the amount of learning that can come from a trial without always dramatically multiplying the cost. They can answer implementation science‐based questions that are broader than the clinical disease and are broadly relevant to implementation in hospitals, clinics, and wider services.

## TYPES OF HYBRID TRIALS

**Figure 1:**
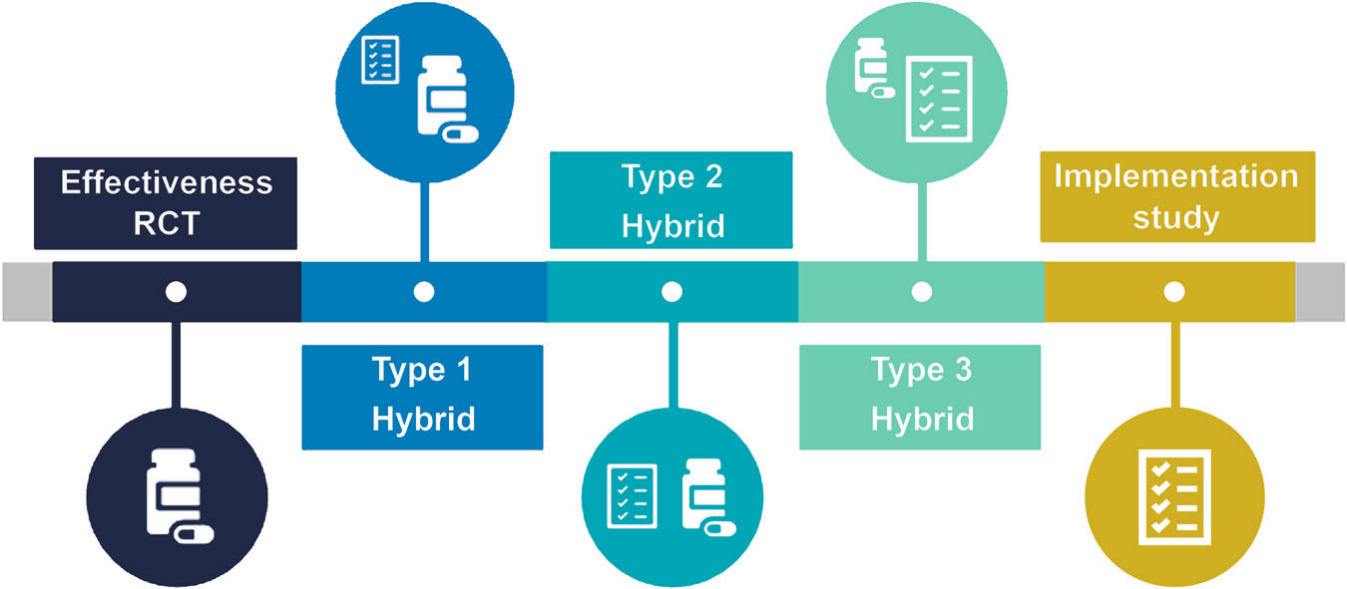
Continuum of hybrid trial designs.^11^

Hybrid trials exist on a continuum, with the three designs varying based on their primary focus and the amount of emphasis on effectiveness versus implementation outcomes (see Table 1 and Figure 1)^11^ ^,^ ^12^:

− A *Type 1 hybrid trial* focuses primarily on the intervention effectiveness outcomes while exploring the context for future intervention implementation (e.g., Wooldridge et al.'s^10^ evaluation of nonadherence in patients who received discharge medication counseling by a pharmacist as part of an RCT; Naef et al.^13^ evaluation of a multicomponent family support intervention in intensive care settings).
− A *Type 2 hybrid trial* has a dual focus on intervention effectiveness and implementation outcomes (e.g., Gilmartin et al.'s^14^ evaluation of the effectiveness of the rural transitions nurse program; Hassett et al.'s^15^ implementation of patient‐reported outcomes for symptom management in oncology practice).
− A *Type 3 hybrid trial* focuses primarily on implementation outcomes while also exploring effectiveness outcomes as they relate to uptake, integration, and fidelity of the intervention in real‐world settings (e.g., Bonafide et al.'s^16^ evaluation of a safety huddle‐based intervention; Salloum et al.'s^17^ evaluation of clinical decision support for patient‐centered chronic pain management).

**Table 1:** Spectrum of hybrid clinical trial design characteristics (adapted from Landes and colleagues^11^ and Curran and colleagues^20^)

	Effectiveness RCT	Hybrid Type 1	Hybrid Type 2	Hybrid Type 3	Implementation study
Research aims	*Aim*: Determine the effectiveness of an intervention	*Primary aim*: Determine the effectiveness of an intervention *Secondary aim*: Explore implementation context	*Coprimary aims*: Determine the effectiveness of an intervention Determine the feasibility and/or impact of an implementation strategy	*Primary aim*: Determine the impact of an implementation strategy *Secondary aim*: Explore clinical outcomes associated with implementation	*Aim*: Determine the impact of an implementation strategy
Units of randomization	*Individual* (consumer, patient, clinician) or *Cluster* (clinical unit, family)	*Individual* (consumer, patient, clinician) or *Cluster* (clinical unit, family)	*Individual* (consumer, patient, clinician) or *Cluster* (clinical unit, facility, system)	*Cluster* (clinical unit, facility, system)	*Cluster* (clinical unit, facility, system)
Comparison conditions	Placebo, treatment as usual, competing intervention	Placebo, treatment as usual, competing intervention	Placebo, treatment as usual, competing intervention	Historical practice or treatment as usual	Historical practice or treatment as usual
Population and sampling framework	One population studied, with strict inclusion/exclusion criteria	Two populations studied *Primary*: Strict inclusion/exclusion criteria *Secondary*: Clinicians, surrounding services	Two populations studied, including one with strict inclusion/exclusion criteria; one examining clinicians and surrounding services	Two populations studied *Primary*: Whole system *Secondary*: Strict inclusion/exclusion criteria	One population studied, only focussing whole system rather than individuals
Measurement and outcomes	Quantitative measures: Clinical effectiveness ± cost	*Primary*: Quantitative measures: Clinical effectiveness ± cost *Secondary*: Mixed methods (interviews, surveys, audits): Feasibility, barrier/enablers to implementation, acceptability of the intervention, sustainability potential	*Coprimary*: Quantitative measures: clinical effectiveness ± cost Adoption and fidelity to clinical treatment (and related factors)	*Primary*: Adoption and fidelity to clinical treatment (and related factors) *Secondary*: Quantitative measures: clinical effectiveness	Adoption and fidelity to clinical treatment (and related factors)

*Note*: Cluster randomization may include traditional cluster‐randomized designs as well as stepped‐wedge cluster‐randomized designs.

## LIMITATIONS

Hybrid trials present their own challenges, which mostly center on complexity. It is vital that research teams include methodological advisors, including clinical trialists and implementation scientists (which may be the same person). Ethics and institutional review board reviews can be difficult, with multiple types of participants and designs for approval. These trials can be more costly at the first granting proposal stage but are arguably less expensive than doing two or three studies to meet the same goals. There is a similar increase in measurement burden at a site level. To mitigate this, Type 3 hybrid trials effectiveness outcome data may need to rely on a subsample of patients, medical record review or administrative data, reducing reliability. Dual goals can cause competing interests, where resources are diverted to ensure effective goals are met, which might deprioritize implementation outcomes. Type 1 hybrid trials may struggle to get buy‐in for participation by clinicians to examine future implementation, without the evidence to suggest effectiveness. These limitations and challenges can be mitigated with a strong and collaborative interdisciplinary team that responds to the constantly shifting implementation context throughout the trial.

Exemplars are now available in the hospital medicine research domain, but the Enhancing the QUAlity and Transparency Of health Research (EQUATOR) network has not developed or registered a guideline for hybrid trials, or any of the subdesigns. For now, researchers need to rely on both the Consolidated Standards of Reporting Trials (CONSORT)^18^ (and subtypes) and the Standards for Reporting Implementation Studies (StaRI) Statement.^19^ Consistency in design and reporting are vital to ensure the maturation of this relatively new design format and to ensure the benefits make it from the trial to the next bedside.

## THE FUTURE

In the future, all “good” trials will be hybrid, in some way. Delays in the traditional research pipeline cause patients to receive suboptimal care, which is unacceptable and fixable. However, resources need to be developed to ensure hybrid trials are achievable and rigorous in their execution. Dedicated funding streams are necessary to both fund high‐quality hybrid trials and advance their methodological science. We hope that this methodological progress note increases awareness of the opportunities that this trial design presents and galvanizes those doing effectiveness trials to consider incorporating these approaches into their own work.

## CONFLICT OF INTEREST

Dr. Beidas receives royalties from Oxford University Press, consulting fees from United Behavioral Health and OptumLabs, and serves on the advisory boards for Optum Behavioral Health, AIM Youth Mental Health Foundation, and the Klingenstein Third Generation Foundation outside of the submitted work. The remaining authors declare no conflict of interest.
